# Surface properties of direct restorations and adjacent dental tissues after saliva exposure

**DOI:** 10.1590/1807-3107bor-2025.vol39.091

**Published:** 2025-09-08

**Authors:** Patrícia Valéria Manozzo KUNZ, Roberta da VEIGA, Vania CAMARGO, Marina da Rosa KAIZER, Gisele Maria CORRER, Carla Castiglia GONZAGA

**Affiliations:** (a) Universidade Positivo, School of Health Sciences, Graduate Program in Dentistry, Curitiba, PR, Brazil.

**Keywords:** Dental Enamel, Dentin, Hardness, Saliva

## Abstract

This study assessed the effect of saliva exposure on roughness (Ra) and Vickers hardness (VHN) of two direct restorative materials, enamel, and dentin adjacent to the restorations. Enamel and dentin cavities in molars (n = 10) were restored with a) bulk-fill resin composite (Tetric N-Flow Bulk Fill, BF) with the application of a universal adhesive (Tetric N-Bond Universal) and b) alkasite restorative material (Cention N, CN) with and without the application of a universal adhesive. After 24 h (baseline), surface roughness and hardness of the restorative material and dental tissues were assessed at 100 μm from the tooth/restoration interface. The specimens were subjected to degradation in whole saliva for 7 days (combined with the application of 20% sucrose 10x/day). Ra and VHN were then reassessed. The data were analyzed by ANOVA and Tukey’s test (α = 5%). BF exhibited a significantly lower Ra than CN. Ra increased significantly after degradation in saliva when compared to baseline values. Enamel hardness was higher for BF restorations. No differences in enamel hardness were observed for alkasite restorations with and without adhesive application. For dentin hardness, significantly lower values were found in alkasite restorations with previous application of an adhesive system. It can be concluded that BF had a smoother surface than CN before and after degradation. Immersion in saliva reduced the hardness of both restorative materials, enamel, and dentin adjacent to the restoration.

## Introduction

Resin composites for posterior restorations have been widely used in daily clinical practice.^1-3^ Besides esthetics, clinicians seek simpler and quicker restorative procedures, often achieved with bulk-fill resins that can be placed in 4- to 5-mm increments.^4, 5^ Bulk-fill composites reduce polymerization shrinkage, decrease stress at the interface, allow light to get through the restorative material, and minimize poor polymerization or the formation of gaps at the interface.^1, 3, 6, 7^ However, concerns about the longevity of esthetic posterior composite restorations have led researchers to evaluate the performance of conventional and bulk-fill materials in terms of fracture, sensitivity, retention, and recurrent caries.^8-12^

Alkasite-based composites (Cention N, Ivoclar Vivadent) have been developed as an alternative for the direct amalgam restoration of posterior teeth and those made with glass ionomer cement or resin composite.^13^ This material contains inorganic fillers (glass fillers, 0.1 to 7 µm) and an isofiller (Tetric-N-Ceram technology). The isofiller particles consist of ground pre-polymerized organic monomers, included as an extra filler in the material, and their particle size ranges from 0.1 to 35 µm. This alkasite composite can be used for class I and II restorations, using a single-increment placement approach, but the previous application of an adhesive system and light-curing is optional.^14^

Alkasites can be classified as a subgroup of restorative composites with alkaline particles that neutralize acidic ions and may remineralize dental tissues.^14^ When adhesive systems are not previously applied, retentive preparations should be used, similar to those used for amalgam. When adhesive systems are applied, the preparation should be less invasive, preserving as much healthy tooth structure as possible.^15^ In restorations without adhesive layer application, it is important to assess the enamel and dentin adjacent to the restorative material, given that an open interface without a hybrid layer can cause failure in the protective sealing of the interface, facilitating microinfiltration.^8^ On the other hand, the open interface for restorative materials such as the alkasite, which have a potential for ion release and ion exchange with the adjacent tissues, allows for dentin remineralization and demineralization prevention.^13,16,17^

Considering that the oral cavity is complex, owing to the presence of saliva (flow, buffer capacity, protein concentrations, etc.), water, and acidic metabolites (with constant changes in pH) produced by the cariogenic biofilm (amount of biofilm accumulation, diversity of species in its composition), retention of restorative materials poses a great challenge.^18-20^ When assessing the properties of restorative materials, surface roughness may be related to the amount of biofilm accumulation, facilitating bacterial adhesion.^21,22^ The polishing capacity and surface roughness of composites are correlated with their particle sizes; the larger the number of large-sized particles in the composite, the worse the polishing capacity and the rougher the surface. Therefore, studies that assess the surface degradation of direct and indirect restorative materials are important to elucidate their physical and mechanical properties, rendering restorative procedures safer, more predictable, and long-lasting.^8,20,23^The present study evaluated the effects of saliva exposure on the surface roughness and hardness of a bulk-fill resin composite and an alkasite-based restorative material. Also, the hardness of the enamel and dentin adjacent to the restorations was assessed. The null hypotheses stated that a) both materials would have a similar roughness and hardness, and b) the hardness of the enamel and dentin adjacent to both restorations would not be different.

## Methods

Twenty healthy human molars free of any carious lesions that had been recently extracted were obtained by direct donation (CAAE 44167421.4.0000.0093, approval protocol no. 4.594.456). The teeth were cleaned and placed in neutral 0.5% chloramine T solution for disinfection. Until their use, the teeth were kept under refrigeration (4 ^o^C) in distilled water, which was changed every week.

The roots were sectioned 1 mm beyond the cementoenamel junction and the crowns were sectioned mesiodistally with a high-precision saw using a water-cooled diamond disc (IsoMet 1000, Buehler, Lake Bluff, USA) for exposure of the flat areas of dentin and enamel. The exposed surfaces were polished with a 600-grit sandpaper for 1 min, rinsed with water spray, and dried. Cavities measuring approximately 2 x 3 x 1.5 mm were made in the hemi-crowns at the enamel/dentin junction, thus including both dental tissues. The preparations were made with a conical round diamond bur (FG 2135, KG Sorensen, Cotia, Brazil) under water cooling. The diamond burs were changed every five cavity preparations.

The hemi-crowns with the preparations were randomly distributed into three experimental groups (n = 10). The cavities were restored with one of the following restorative materials, following the manufacturer’s instructions: a) bulk-fill resin composite (Tetric N-Flow Bulk Fill, Ivoclar Vivadent, Schaan, Liechtenstein) with previous application of a universal adhesive (Tetric N-Bond Universal, Ivoclar Vivadent Saliva) or b) alkasite composite (Cention N, Ivoclar Vivadent, Schaan, Liechtenstein) with and without prior application of the universal adhesive. In the groups in which the adhesive was applied, the procedure was standardized with the application of two consecutive layers, rubbing the adhesive on the surfaces for 10 s, followed by a light air jet. The adhesive layer was light-cured for 20 s. For Cention N, a homogeneous mix was obtained by combining one measuring scoop of powder with one drop of liquid, ensuring a 4.6:1 weight ratio. The material was mixed for 45-60 s to achieve a workable consistency. The material was then applied to the cavity, carefully adapted, condensed, and excess material was removed. The resin composite was placed in a single increment and light-cured for 40 s with a LED light curing unit at an irradiance of 1,200 mW/cm^2^ (Demi Led, Kerr, Orange, USA). The specimens were finished and polished with aluminum oxide-impregnated discs (Sof-Lex, 3M Oral Care, St. Paul, USA) for 10 s each, without water cooling. The restored teeth were stored in distilled water at 37ºC for 24 h (Table 1).

**Table 1:** Composition of the assessed restorative materials.

Restorative material	Composition	Application mode
Tetric N-Flow Bulk Fill	Matrix: Bis-GMA, UDMA, TEGDMA	1. Application of Tetric N Bond Universal adhesive
	Load: barium glass, ytterbium (III) fluoride, mixed oxide, silicon dioxide	2. Increments up to 4 mm in depth, light-cured for 10 s at an irradiance of 1,200 mW/cm^2^
	Filler: 46.4 vol% / 68.2 wt%	
Cention N	Powder: barium-aluminum silicate glass, ytterbium (III) fluoride, isofiller, calcium aluminum fluorosilicate glass, calcium fluorosilicate glass.	1. Application of Tetric N Bond Universal adhesive
	Liquid: UDMA, DCP, tetramethyl-xylylene-diurethane dimethacrylate, PEG-400 DMA	2. Handling of the material in the following amounts: 2 measuring scoops of powder and 2 drops of the liquid (corresponding to a weight of 4.6:1)
		3. Light-curing for 20 s for cavities larger than 3 mm in depth, at an irradiance of 1,200 mW/cm^2^
	Filler 57.6 vol% / 78.4 wt%	

Bis-GMA: bisphenol A-glycydil methacrylate, UDMA: urethane dimethacrylate, TEGDMA: triethylene glycol dimethacrylate, DCP: dicalcium phosphate, PEG-400 DMA: polyethylene glycol-400 dimethacrylate.

Surface roughness was assessed with a roughness tester (Surftest SJ-210, Mitutoyo Corporation, Kawasaki, Kanagawa, Japan) and a needle at a constant speed of 0.5 mm/s and 0.7 mN load. Ra (roughness average) values for each specimen were measured using a standard length of 0.25 mm. The roughness of the restorative materials was evaluated in the central region of the restorations to avoid interference with the measurements. Surface roughness was measured in two directions on each restoration: mesiodistally and occlusocervically. The average of these measurements was then calculated and used for statistical analysis to provide a comprehensive assessment of the surface characteristics of the restorations.

Vickers hardness was measured with a microhardness tester (HM-210, 810-404A, Mitutoyo Corporation, Kawasaki, Kanagawa, Japan) with a load of 200 g and a dwell time of 15 s. For calculation of the hardness of the restorative material, three measurements were made for each specimen and the mean of the indentations was used to estimate the Vickers hardness number (VHN). Hardness was assessed in a different area from that used for roughness measurement, such that indentations would not interfere with roughness measurements.

Ten healthy adults aged 18 to 25 years were selected according to the following criteria for good systemic and oral health: no use of medications that could alter the bacterial flora and salivary consistency, nonuse of orthodontic appliances or denture, normal salivary flow, no sign of gingivitis or active caries, and compliance with the study. The volunteers who were eligible for the study and who agreed to participate signed an informed consent form.

Passive whole saliva was collected from healthy volunteers without any external stimulation. Saliva was collected in sterile cups at a standardized time (9h00) to minimize diurnal variations in salivary flow. Participants were instructed to maintain good oral hygiene prior to saliva collection to reduce contamination. The saliva was stored in containers labeled with the donors’ data. A drop of 20% sucrose was applied to the storage solution 10 times a day at 8h00, 9h30, 11h00, 12h30, 14h00, 15h30, 17h00, 18h30, 20h00, and 21h30. The hemi-crowns were placed individually in a lidded sterile container and stored in the whole saliva collected from the volunteers. The specimens were stored at 37ºC for 7 days, and the saliva was replaced every 2 days through a new collection.^20^ After 7 days, the specimens were reassessed for surface roughness and hardness.

Surface roughness [specimens after 24 h of storage (baseline) and after degradation in saliva (7 days)] was evaluated, as described earlier. Vickers hardness of the enamel or dentin adjacent to the restorations was also assessed. Indentations were made at 100 μm on the subsurface of the enamel and dentin from the tooth/restoration interface.

Shapiro-Wilk and Levene’s tests were used to assess data normality and homogeneity of variances, respectively. The roughness and hardness data for the restorative materials and the enamel and dentin adjacent to the restorations were analyzed by repeated-measures two-way ANOVA and Tukey’s test. All analyses were performed with significance set at 5% using the SigmaPlot 11 software (Systat Software, Inc., San Jose, USA). Post-hoc power analysis was performed, and observed power values between 0.835 and 1 were obtained.

## Results

Surface roughness showed statistically significant differences for restorative material (p < 0.001) and time (p = 0.024); the double interaction was not significant (p = 0.303). Bulk-fill resin exhibited significantly lower Ra than alkasite. By comparing the results for 24 h and 7 days, degradation in saliva yielded significantly higher Ra than at baseline (Table 2).

**Table 2:** Mean and standard deviation of roughness average (Ra) of the assessed restorative materials before and after saliva immersion.

Restorative material	Ra (μm)		
	24 h	Saliva 7 days	Total
Bulk-fill	0.115 ± 0.007	0.120 ± 0.009	0.118 ± 0.008 a
Alkasite	0.617 ± 0.016	0.629 ± 0.018	0.623 ± 0.017 b
Total	0.366 ± 0.265 A	0.375 ± 0.268 B	

*Values followed by the same uppercase letters in the rows are statistically similar (p > 0.05). Values followed by the same lowercase letters in the columns are statistically similar (p > 0.05).

VHN for restorative materials at baseline and after 7 days in saliva is shown in Table 3. Statistically significant differences were observed for time (p < 0.001); restorative material (p = 0.152) and double interaction (p = 0.357) were not significant. Hardness values were higher at baseline than after 7 days, regardless of the material.

**Table 3:** Mean and standard deviation of Vickers hardness number (VHN) for the assessed restorative materials before and after saliva immersion.

Restorative material	VHN (restorative material)		
	24 h	Saliva 7 days	Total
Bulk-fill	148.4 ± 16.2	142.0 ± 14.5	145.2 ± 15.3 a
Alkasite without adhesive	140.0 ± 13.8	133.7 ± 14.6	136.9 ± 14.2 a
Alkasite with adhesive	134.9 ± 13.5	130.4 ± 13.1	132.6 ± 13.1 a
Total	141.1 ± 15.1 A	135.4 ± 14.4 B	

*Values followed by the same uppercase letters in the rows are statistically similar (p > 0.05). Values followed by the same lowercase letters in the columns are statistically similar (p > 0.05).

For enamel adjacent to the restoration interface (Table 4), there were statistically significant differences for restorative material (p = 0.006) and time (p < 0.001); double interaction was not significant (p = 0.763). Enamel hardness was higher at 24 h when compared to storage for 7 days in saliva. Enamel hardness was significantly higher for restorations with bulk-fill resin; enamel hardness for restorations with alkasite with and without adhesive application was similar between themselves and lower than that of the bulk-fill resin.

**Table 4:** Mean and standard deviation of Vickers hardness number (VHN) for enamel adjacent to the restoration before and after saliva immersion.

Restorative material	VHN (enamel adjacent to restoration)		
	24 h	Saliva 7 days	Total
Bulk-fill	235.1 ± 19.0	230.4 ± 18.6	232.2 ± 17.6 a
Alkasite without adhesive	203.9 ± 2.2	200.1 ± 2.6	202.0 ± 3.0 b
Alkasite with adhesive	207.0 ± 1.3	201.4 ± 4.1	204.2 ± 4.1 b
Total	215.3 ± 17.7 A	210.6 ± 15.5 B	

*Values followed by the same uppercase letters in the rows are statistically similar (p > 0.05). Values followed by the same lowercase letters in the columns are statistically similar (p > 0.05).

Regarding the hardness of the dentin adjacent to the restoration (Table 5), statistically significant differences were observed for restorative material (p = 0.005) and time (p < 0.001); double interaction was not significant (p = 0.104). Higher dentin hardness was observed at 24 h as compared to storage in saliva for 7 days. Dentin hardness was significantly higher for the groups restored with bulk-fill resin and alkasite without adhesive application than for the groups treated with alkasite and previous application of an adhesive system.

**Table 5:** Mean and standard deviation of Vickers hardness number (VHN) for dentin adjacent to the restoration before and after saliva immersion.

Restorative material	VHN (dentin adjacent to restoration)		
	24 h	Saliva 7 days	Total
Bulk-fill	137.1 ± 3.4	134.2 ± 3.5	135.7 ± 3.6 a
Alkasite without adhesive	137.5 ± 18.8	135.7 ± 17.5	136.6 ± 16.8 a
Alkasite with adhesive	106.9 ± 1.8	106.3 ± 1.9	106.6 ± 1.8 b
Total	127.2 ± 18.0 A	125.4 ± 16.9 B	

*Values followed by the same uppercase letters in the rows are statistically similar (p > 0.05). Values followed by the same lowercase letters in the columns are statistically similar (p > 0.05).

## Discussion

Both null hypotheses were rejected in the present study. The observed differences in roughness and hardness between the two restorative materials suggest that material composition and properties influence the degree of surface degradation over time. Additionally, the differences in the hardness of the adjacent dental tissues highlight the potential impact of the restorative materials on the underlying tooth structure. These findings highlight the importance of material selection and clinical technique in achieving long-term restorative success.

Bulk-fill resins and alkasite-based composites have been developed to improve restorative procedures in posterior teeth, using a simplified cavity-filling approach. Alkasites are a subgroup of resin composites and are characterized mainly by the presence of glass particles in their organic matrix that release alkaline ions such as fluoride and calcium, which can neutralize those acidic ions in the vicinity of the restorations.^24,25^ Alkasites appear to be a promising esthetically acceptable restorative material for posterior teeth that require a simple clinical procedure. Several studies have evaluated the properties of bulk-fill resins, but few have assessed alkasites. In the present study, bulk-fill resin exhibited significantly lower Ra values than those of alkasite, both at baseline and after saliva exposure. This discrepancy in surface roughness is likely attributable to several factors, including the ion-releasing properties of alkasite, which contribute to surface degradation over time. The manual mixing technique employed for Cention N may introduce voids and irregularities into the material, further increasing its surface roughness.^13^ Additionally, filler particle size of bulk-fill composites has been estimated to range between 0.1 and 4 μm (with some larger particles around 20 μm),^26^ whereas Cention N has larger isofiller particles ranging from 0.1 to 35 μm.^14^

The decision to use saliva rather than artificial saliva or distilled water as a degradation medium in this study was an attempt to mimic the oral environment, with the presence of salivary enzymes, bacteria, and sucrose. This method was used in a previous study and proved to promote surface degradation of indirect restorative materials.^20^ By exposing the restorations to collected whole saliva, we were able to assess the impact of oral fluids on the surface properties of the material, which is a critical factor in the long-term clinical success of direct restorations. This approach allows for a more realistic evaluation of the material’s behavior in the oral environment. It is well known that resins and glass ionomers can be strongly degraded in the presence of cariogenic biofilm.^27^ In situ studies have already reported that, after 14 days, the consumption of cariogenic foods and biofilm accumulation on the restored surface can speed up the degradation of ionomers, but their influence on resin composites was negligible.^28^ The surface roughness of Cention N and other restorative materials has also been evaluated by two previous studies. Naz et al.^29^ conducted a 3D analysis of roughness before and after 50,000 chewing simulation cycles for a nanohybrid composite, a glass ionomer cement, and alkasite. After the chewing simulation, alkasite surface roughness was lower than that of the other materials. Yazkan^30^ assessed the effects of energy drinks and soft drinks on the surface degradation of several direct restorative materials, such as alkasite, high-viscosity glass ionomer cement, and glass carbomer. In that study, Cention N exhibited a smoother surface than that of glass ionomer cement.

The present study revealed lower hardness of the assessed materials, enamel, and dentin adjacent to the restoration after degradation in saliva for 7 days. Also, the hardness of the dentin adjacent to the bulk-fill resin and alkasite without an adhesive application was higher than that of the dentin restored with alkasite and adhesive layer. In the case of dentin and alkasite, the adhesive can negatively influence the hardness and other properties, acting as a barrier and decreasing mineralization and the incorporation of ions released by the material. While the hardness of dentin adjacent to Cention N restorations when used with an adhesive system should not be considered in isolation, the clinical implications of this combination are noteworthy. When bonded to tooth structure using an adhesive system, Cention N demonstrates bond strength and microleakage properties comparable to those of conventional resin composites. This finding underscores the significance of utilizing an adhesive system in conjunction with Cention N to achieve optimal clinical outcomes.^31^

The effectiveness of bioactive ion-releasing materials (glass ionomer, glass hybrids, calcium silicate cement, and alkasite) in repairing artificially demineralized dentin lesions was previously assessed. It was demonstrated that all evaluated materials led to an increase in microhardness and affected the aspect and chemical composition of the dentin surface, with the glass hybrid EQUIA Forte HT having the most significant impact.^32^ Other studies have assessed fluoride release by alkasite, comparing it with conventional glass ionomer cement and resin composite.^16, 24^ In these studies, alkasite was evaluated with and without the use of adhesive systems in the cavity, and with or without light-curing. In the present study, Cention N was light-cured, given that it was compared with a bulk-fill resin. Alkasite provided good outcomes, with a lower incidence of marginal infiltration in the chemically activated polymerization group, without an adhesive system.^16^ In the study by Kelić et al.,^17^ the amount of released fluoride ions varied between the dental materials assessed and relied upon adhesive systems and surface coatings. Glass ionomer cement showed the highest amount of fluoride ion release, followed by alkasite and giomer. Adhesive systems and surface coatings reduced the release of fluoride ions. Mesham et al.^24^ found that the use of adhesives in the cavity and photoactivation of the alkasite resin showed similar outcomes to those of the resin composite. It is important to highlight, however, that the use of adhesive systems with alkasite has demonstrated positive results related to bond strength when compared to glass ionomer cements and resin composites.^15,29^

While the previous use of adhesive systems has been positive for bond strength when alkasite is used in non-retentive cavities, the possibility of restoring class I and II cavities with a self-adhesive material is clinically relevant, showing a growing trend in the search for self-adhesive direct restorative materials that can be placed in larger increments or with single placement in posterior teeth.^15,18,33,34^ Currently, there are commercially available experimental versions of self-adhesive bulk-fill resins, which have been assessed to ensure the positive aspects of alkasite in materials with better mechanical properties.^15,31,35^

A randomized clinical trial study compared Cention N (without the use of adhesive) and resin composite class II restorations over one year. Restorations using CN and resin composite had survival rates of 92.5% and 97.7%, respectively. There were no significant differences for surface texture, marginal adaption, loss of retention, and marginal discoloration. None of the restorations showed signs of secondary caries or post-operative sensitivity. After 12 months, both restorative materials demonstrated comparable clinical performances.^36^

Due to its versatile use and good mechanical properties, the alkasite-based material can be a suitable alternative to glass ionomer cement. One clinical limitation of this material is that it has lower viscosity at higher temperatures, which could interfere with the shaping and sculpting of the restoration. Notwithstanding the positive features of the clinical use of alkasite, improvement in the consistency and rheological properties of the material may facilitate sculpting, mainly when used under non-optimal or controlled conditions. While this study provides evidence of the behavior of direct restorative materials under simulated oral conditions, it is important to acknowledge its limitations. The use of whole human saliva, while more clinically relevant than artificial saliva, may introduce variability due to individual differences in salivary composition. Additionally, the short aging period employed in this study, while providing a practical approach to evaluate material degradation, may not fully capture the long-term effects of oral exposure. Furthermore, the study focused on surface properties and did not assess other important factors, such as mechanical properties and color stability.

## Conclusion

It can be concluded that:

- restorative material and saliva immersion influenced surface roughness;
- bulk-fill resin showed a smoother surface than that of alkasite under all conditions;
- immersion in saliva reduced hardness of both materials, enamel, and dentin adjacent to the restorations
- enamel and dentin adjacent to the restoration had higher microhardness at the bulk-fill/restoration interface
- regarding alkasite, the application of an adhesive system before the placement of the restorative material impaired the hardness of the dentin adjacent to the restoration.

**Figure:**
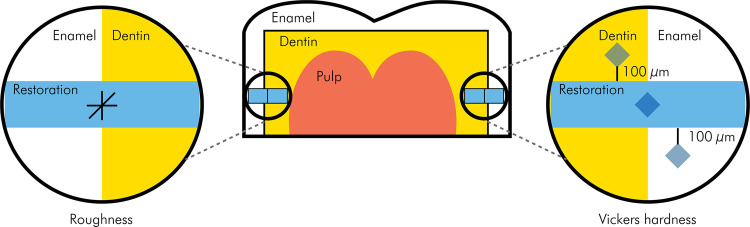
Schematic diagram of hemi-crowns with restored cavities for assessment of surface roughness of restorative materials and Vickers hardness of materials and tooth structure adjacent to the restoration before and after saliva exposure.

## Data Availability

The datasets generated during and/or analyzed during the current study are available from the corresponding author on reasonable request.
